# Biological function of Presenilin and its role in AD pathogenesis

**DOI:** 10.1186/2047-9158-2-15

**Published:** 2013-07-17

**Authors:** Shuting Zhang, Mingming Zhang, Fang Cai, Weihong Song

**Affiliations:** 1 Townsend Family Laboratories, Department of Psychiatry, Brain Research Center, Graduate Program in Neuroscience, The University of British Columbia, 2255 Wesbrook Mall, Vancouver, BC V6T 1Z3, Canada

## Abstract

Presenilins (PSs) are the catalytic core of γ-secretase complex. However, the mechanism of FAD-associated PS mutations in AD pathogenesis still remains elusive. Here we review the general biology and mechanism of γ-secretase and focus on the catalytic components – presenilins and their biological functions and contributions to the AD pathogenesis. The functions of presenilins are divided into γ-secretase dependent and γ-secretase independent ones. The γ-secretase dependent functions of presenilins are exemplified by the sequential cleavages in the processing of APP and Notch; the γ-secretase independent functions of presenilins include stabilizing β-catenin in Wnt signaling pathway, regulating calcium homeostasis and their interaction with synaptic transmission.

## Introduction

Alzheimer’s disease is the most common neurodegenerative disorder leading to dementia, accounting for two thirds of dementia in elderly populations. The majority of AD cases are late-onset and sporadic without defined causes, whereas less than 5% of cases are familial with early-onset and caused by gene mutations. Genetic studies have shown that four genes confer susceptibility to AD: amyloid-β precursor protein (APP) on chromosome 21 [1-6], Presenilin 1 (PS1) on chromosome 14 [7-10], Presenilin 2 (PS2) on chromosome 1 [11-13] and apolipoprotein E (ApoE) on chromosome 19 [14,15]. Neuritic plaques, neurofibrillary tangles (NTFs) and neuronal loss are pathological hallmarks of AD. However, the mechanism underlying AD pathogenesis remains elusive and there is no effective prevention or treatment to this devastating disorder so far.

Neuritic plaques are formed by extracellular deposits of amyloid β protein (Aβ) [16]. Aβ is derived from proteolytic processing of APP and consists primarily of 40- and 42-amino acid residues, with the more hydrophobic Aβ42 as the major component in neuritic plaques [16,17]. NFTs are intraneuronal inclusions composed of hyperphosphorylated forms of the microtubule-associated protein Tau [18-21]. Aβ-containing neuritic plaques are the unique pathological feature in AD brains whereas NTFs could also be detected in other dementia subtypes like frontotemporal dementia with Parkinsonism caused by mutations on MAPT gene [22]. Current prevailing “amyloid hypothesis” in AD suggests that the accumulation of Aβ, particularly the more hydrophobic and aggregation-prone Aβ42, being soluble oligomers [23-29] or aggregate fibril form, initiates neuronal dysfunction, resulting in neurodegeneration in AD [30].

**Figure 1:**
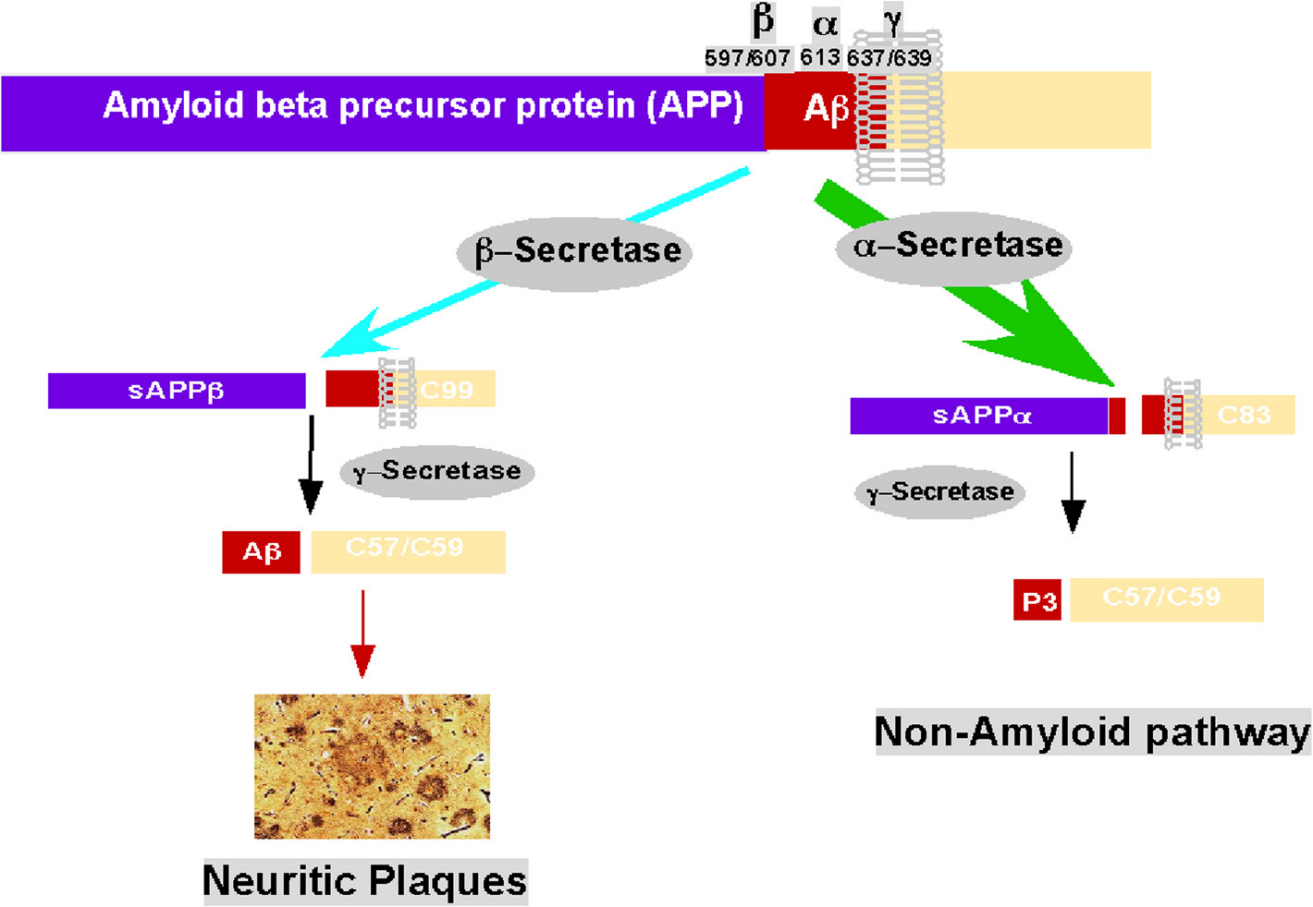
**APP processing pathways.** Under the physiological conditions, the majority of APP undergoes the non-amyloidogenic pathway. The α-secretase processes APP within the Aβ domain to generate C83 and this cleavage abolishes Aβ generation. In the amyloidogenic pathway, β-secretase processes APP at Asp^1^ site to generate C99 fragment, which is the substrate for γ-secretase for Aβ generation.

The central event of “amyloid hypothesis” is APP processing. APP undergoes posttranslational proteolytic processing by α, β and γ-secretases (Figure 1). The majority of APP is constitutively processed by α-secretase within the Aβ domain in a non-amyloidogenic pathway [31]. In the amyloidogenic pathway, APP undergoes sequential cleavages by β- and γ-secretase to generate Aβ. A transmembrane aspartic protease BACE1 was identified as β-secretase [32-35]. BACE1 processes APP at the Asp^1^ site of Aβ domain to generate APP C99 fragment [34,36]. The C99 fragment is further processed by γ-secretase at the intramembrane Val^40^ and Ala^42^ sites to generate Aβ40 and Aβ42, respectively. The second cleavage, which takes place within the hydrophobic transmembrane domain (TMD) and is termed as regulated intramembrane proteolysis (RIP) [37], has been attributed to γ-secretase with presenilins as the catalytic component [38-45].

As the catalytical component of γ-secretase, the first part of this review will focus on the contribution of presenilins to γ-secretase and its role in AD pathogenesis in the scenario of “Amyloid hypothesis”. The rest of this review will discuss diverse biological functions of presenilins independent of γ-secretase activity. Its well-established role in β-catenin/Wnt-signaling and calcium homeostasis as well as the contribution to the AD pathogenesis will be addressed.

## Presenilins and γ-secretase

### Presenilins

Presenilins have two homologs, PS1 and PS2, with 67% identical sequence [11]. mRNAs of both Presenilins are ubiquitously detected in many human and mouse tissues, including brain, heart, kidney and muscle [46]. PS1 and PS2 are highly conserved and functionally redundant with SEL-12 as their homolog in *Caenorhabditis elegans*[47].

**Figure 2:**
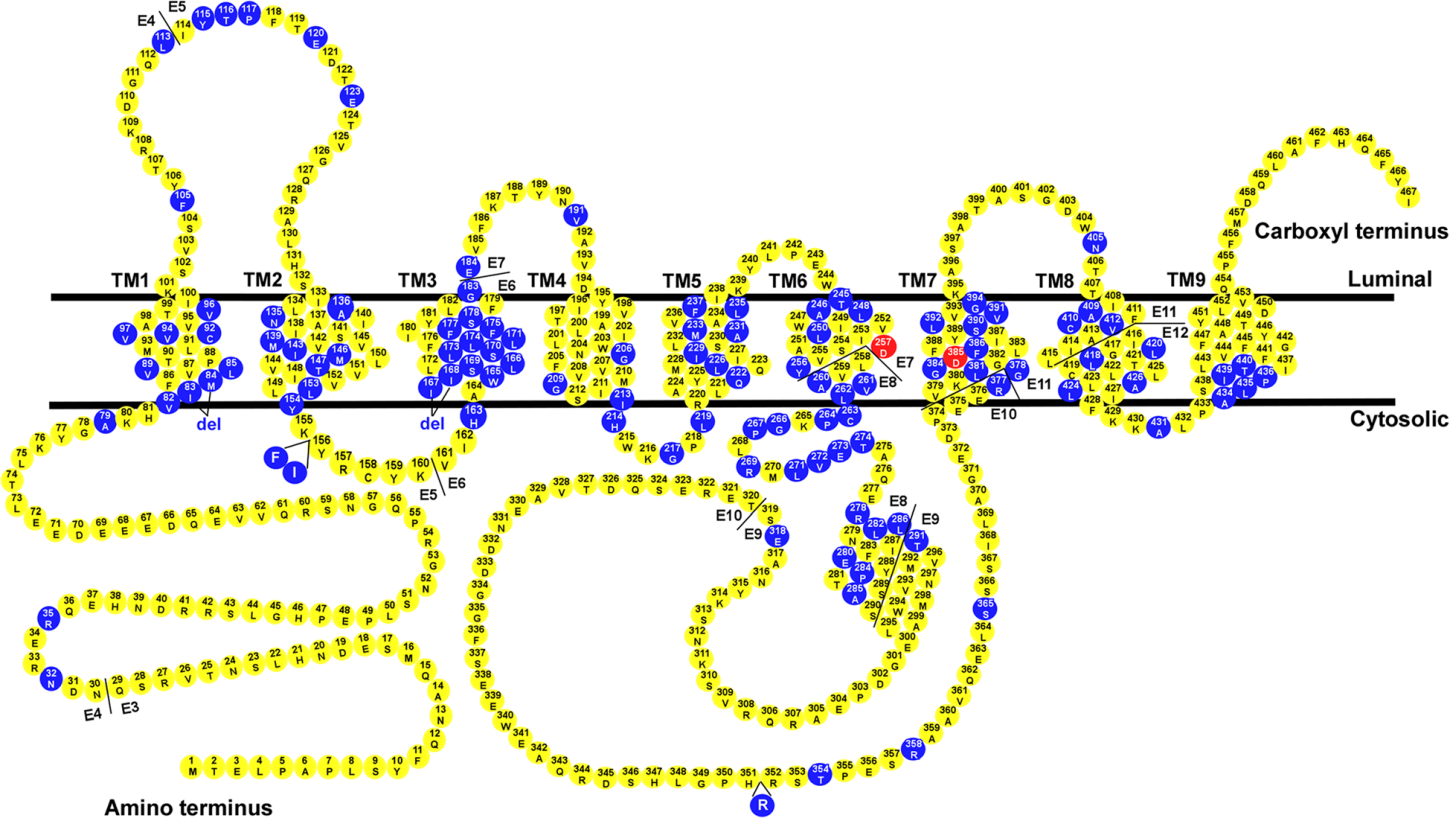
**Presenilin 1 structure.** This diagram shows the amino acid sequence of PS1 and the distribution of the FAD-associated mutations. Blue circles represent the FAD-associated mutations and red circles indicate the two catalytic active aspartates.

PS1 is a multi-transmembrane protein with nine-transmembrane topology (Figure 2) [48,49], and abundantly present in the ER and trans-Golgi network [50-53]. Under physiological condition, the majority of PS1 undergoes endoproteolysis within the large hydrophobic loop in the cytoplasmic side to generate N-terminal fragment (NTF) and C-terminal fragment (CTF) [54]. The endoproteolytic cleavage takes place at heterogeneous sites from amino acid 292 to 299 [55-57]. While some studies reported an independent protease as the “presenilinase” [58,59], growing evidence supported the hypothesis that PS undergoes autoendoproteolysis [43,60-64]. The endoproteolysis event might be important to render the γ-secretase activity to PS NTF/CTF heterodimer by removing the auto-inhibitory effect of the large hydrophobic loop [64,65]. However, it is not clear whether endoproteolysis is an absolute requirement for the maturation of presenilins since some presenilin mutants are enzymatically active in the absence of endoproteolysis, as are the cases in FAD-associated PS1ΔE9 and PS2 M292D [57,66].

### γ-Secretase complex assembling

γ-secretase is essential for cleavage of APP C99 to generate Aβ [67]. γ-secretase is a multi-unit enzymatic complex, including presenilin NTF/CTF heterodimer, nicastrin, Aph-1 and Pen-2 [39,41-45]. Presenilins are the first molecules identified to be associated with γ-secretase *in vivo* and *in vitro*. PS1 knockout mice showed markedly reduced γ-secretase cleavage of APP [38] and knockout of both PS1 and PS2 completely abolished γ-secretase activity [40,68]. Using anti-PS antibody, Yu et al. identified Nicastrin, an integral transmembrane protein with a large N-terminal domain, as the second γ-secretase component [39]. However, expression of both presenilins and nicastrin don’t suffice to restore γ-secretase activity, indicating the existence of other components. Further gene screening studies on the *glp*-1 (Notch homolog) deficient phenotype of *C.elegans* discovered Aph-1 and Pen-2 as another two components of γ-secretase [41,42]. Aph-1 is a 30 kDa multi-transmembrane protein like presenilin, whereas Pen-2 is a 12 kDa hairpin-like transmembrane protein. Co-expression of presenilin, Aph-1, Pen-2 and nicastrin increases γ-secretase activity in transfected cells and the four proteins together are sufficient to reconstitute γ-secretase activity in yeast [44,69].

Previous studies demonstrated that the minimal molecular weight of γ-secretase complex was around 200–250 kDa, implying a 1:1:1:1 stoichiometry of PS/Nicastrin/Aph-1/Pen-2 in γ-secretase complex [44]. Though it was accepted that the four molecules were the minimal γ-secretase complex assembling, recent report suggested that the PS/Pen-2/Aph-1 complex was sufficient for the catalytic activity in the absence of Nicastrin [70]. Another study demonstrated that PS1ΔE9 alone had partial γ-secretase activity and PS1ΔE9/Pen-2 was sufficient to restore full γ-secretase activity [71]. These studies suggest the complexity of γ-secretase complex assembling. Given the stoichiometry of γ-secretase complex and the existence of PS and Aph-1 homologs, there are at least six different forms of γ-secretase complex that could be assembled [72,73]. PS1-involved complex or PS2-involved complex processed APP C99 differentially and showed distinct susceptibility to certain γ-secretase inhibitors [74,75], indicating different γ-secretase complexes with possible distinct functions.

### Structure of γ-Secretase complex

**Figure 3:**
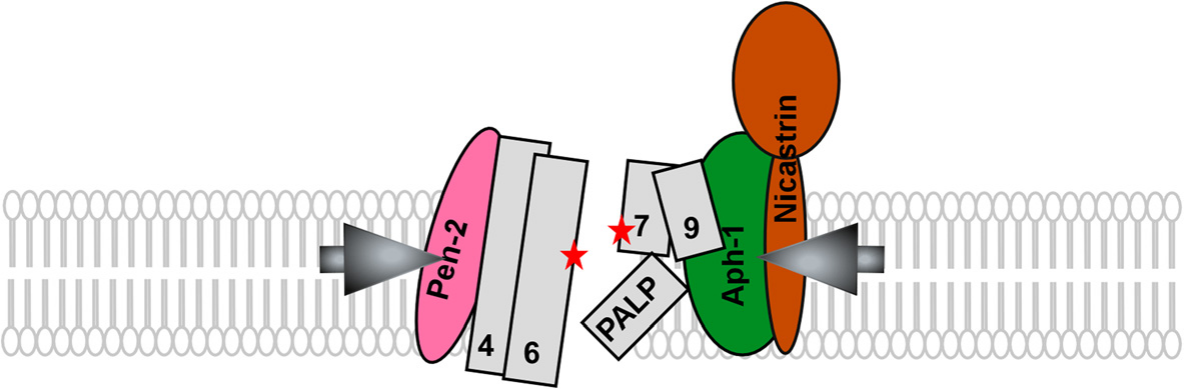
**γ-secretase complex and the formation of the catalytic pore of PS1.** PS1 transmembrane domains (TMDs) are shown as columns with numbers. Without the assistance of other subunits, PS1 forms a relatively open pore structure within the membrane. Upon the binding of subunits, the PALP motif moves to the proximity of the catalytic center, and the catalytic structure is activated by the structural changes in TMDs of PS1.

The catalytic core of γ-secretase complex is presenilins. Presenilins, together with signal peptides peptidases (SPPs), belong to aspartyl intramembrane cleaving proteases (I-CLiPs) [76]. The two catalytic aspartate residues (Asp^257^ in transmemberane 6 (TM6) and Asp^385^ in TM7) are located at NTF and CTF of presenilins, respectively. Mutations on either aspartate abolish the enzymatic activity of γ-secretase complex [60]. With a large highly glycosylated ectodomain, nicastrin has been implicated to function as the initial recognition of substrates [77]. Electronic microscopic analysis and single particle imaging revealed the existence of intramembrane water-accessible cylindrical chamber in gamma-secretase with a low-density cavity from extracellular side [78,79]. Parallel substituted cysteine accessible method (SCAM) and cross-link experiment confirmed that TM6, TM7 and TM9 of PS formed the intramembrane chamber with two catalytic aspartates residing oppositely on TM6 and TM7, respectively [80-84]. The constitutive autoendoproteolysis of PS removes the inhibitory allosteric effect of the large hydrophobic loop from the catalytic chamber structure in PS [64,65]. With direct interaction between γ-secretase components [85,86], Nct/Aph-1 subunits and Pen-2 tighten the relative loose PS TM6/TM7/TM9 intramembrane cavity and rearrange the PALP motif of TM9 to the proximity of the catalytic center, thus activate the γ-secretase complex [87] (Figure 3). Recently, Li et al. reported the crystal structure of a presenilin/SPP homologue (PSH) from the archaeon Methanoculleus marisnigri JR1 and predicted the structure of presenilin based on the conserved sequence between the two homologues [88]. They confirmed the existence of the water permissible cavity but also revealed some differences in TM7 and TM9 compared with the NMR structure of PS1 CTF. The work shed new light on elucidation of the crystal structure of presenilin.

### γ-Secretase substrates and sequential cleavages

#### APP and Notch as classical substrates

γ-Secretase preferentially processes type I integral membrane protein after the ectodomain is shedded [89]. It is intriguing that many of its classical substrates function in the signaling pathways, cell adhesion and migration, neuritis outgrowth and synapse formation, and many of these events are often disrupted during AD pathogenesis [90]. The number of substrates is growing to over 80, including APP, Notch, neuregulin, ErbB4, E-cadherins and N-cadherins, CD44 and growth hormone receptor [40,91-98].

APP and Notch are two most well known γ-secretase substrates. γ-Secretase is named after it function as the enzyme to process APP at the γ-cleavage site to generate Aβ, which is currently believed to play an essential role in the “Amyloid cascades” in AD pathogenesis. Notch is a type I transmembrane cell surface receptor that mediates cell fate decisions in both vertebrates and invertebrates [99,100]. After it is cleaved by furin, cell surface Notch receptor heterodimers bind to the DSL (Delta/Serrate/LAG-2) family ligands on the surface of neighboring cells, then the transmembrane-intracellular fragment of Notch undergoes further proteolysis to release Notch intracellular domain (NICD) from the membrane to the nucleus to activate target genes [101,102]. Presenilins have been shown to play an essential role in Notch signaling. PS-deficient mice exhibit Notch-knockout phenotype [103,104]. Knockout of PS abolishes intramembrane γ-secretase cleavage of Notch as well as the following release of NICD [40,91,105], and FAD-associated PS mutations impair the generation of NICD [91]. Although it is reported that the impaired Notch-signaling is involved in synaptic plasticity and late-onset cognitive decline [106-110], the contribution of Notch- signaling to AD pathogenesis remain to be elucidated.

#### γ-secretase cleavages at ϵ-site and γ-site

γ-Secretase can process substrates at multiple cleavage sites. γ-secretase cleaves the transmembrane domain of APP at two positions: the γ-site to generate Aβ and the downstream ϵ-site to produce the APP intracellular domain (AICD) [111]. Cleavage at the γ-site is heterogeneous, producing Aβ of 39–43 residues, whereas the cutting at the ϵ-site produces AICD of 50 residues almost exclusively. The same phenomenon occurs in Notch processing: heterogeneous cleavages at the S4 site (γ-site) to generate Nβ and homogeneous cleavage at the S3 site (ϵ-site) to generate NICD [112]. Recent independent studies supported the notion that the ϵ-cleavage occurs prior to γ-cleavage [113-115]. Qi-Takahara and colleagues first detected Aβ49, the proteolytic counterpart to AICD_50-99_[113]. Later Ihara and colleagues demonstrated that ϵ-cleavage occurs first and produces Aβ48 and Aβ49 for later γ-cleavage, based on the presence of the induction period for the generation for tripeptides/tetrapeptides detected by liquid chromatography tandem mass spectrometry (LC-MS/MS) in cell-free γ-scretase system [116]. The various Aβ species (ranging from 49- to 40-amino acids) and corresponding tripeptides released from the trimming of Aβ48/49 were identified using LC-MS/MS, further confirming the sequential γ-cleavage from the ϵ-site to γ-site [113,116,117].

#### The effect of FAD-associated presenilins mutations on γ-cleavages

Presenilin mutations are the main cause reasons of early-onset FAD. Presenilin mutations result in the production of the more hydrophobic Aβ42 either in conditioned medium *in vitro* assay [67,118] or in APP/PS1 transgenic mice [119]. It still remains elusive how PS1 mutations affect the enzymatic activity on ϵ- and/or γ-site to initiate the AD pathogenesis. Considering the significant role of Notch in neurogenesis and impaired Notch-signaling in the scenario of presenilins mutations, the contribution of Notch signaling has always been a debating topic in AD field.

γ-Secretase processes its substrates at γ- and ϵ-site, generating distinct products exemplified as Aβ and NICD. Aβ plays central role in AD pathogenesis; whereas NICD is the nuclear transcription factor activator involving in evolutionarily conserved pathway, mediating short-range intercellular communication and cell-fate determination in development as well as in adulthood. Presenilins mutations affect the production of Aβ as well as the generation of NICD, indicating that presenilins mutations influence both γ- and ϵ-cleavages. However, recent studies indicate that γ and ϵ cleavages are distinct enzymatic events with their own enzymatic kinetics and pharmaceutical characterization, and they can be differentially affected by the FAD-associated PS mutations. Fukumori and colleagues reported that the inhibition of endocytosis of PS1 altered AICD formation without changing Aβ42/Aβ40, implying that the efficiency of γ and ϵ cleavage of γ-secretase are different event in plasma membrane and endosome, respectively [120]. Parallel study on TMP21 directly pointed out that TMP21 acted as γ-secretase modulator but affected γ cleavage only [121]. The artificial PS_ΔE10_ impaired the normal Aβ generation but spared the intracellular domain production in both APP and Notch, supporting the possibility that γ- and ϵ-cleavages are dissociated [122]. Using *in vitro* enzyme kinetics assays, De Strooper and his team demonstrated that PS mutations consistently exhibited impaired γ-cleavage activity with altered Aβ42/40 ratio, but the effect on the ICD-producing ϵ-cleavage was varied and substrate-specific. For instance, PS_M139V_ displayed enhanced ϵ-cleavage in N-cadherin but unchanged in APP, Notch and Erb4 [123]. All those studies indicated that the inefficient processing on ϵ-cleavage and the impaired Notch-signaling are not essential for AD pathogenesis.

## Presenilins beyond γ-secretase

Recently, mounting evidence has supported that presenilins carry out multiple functions beyond the catalytic functions of γ-secretase. The conditional knock-out of presenilins in excitatory neurons demonstrated age-dependent neurodegeneration, indicating an essential role of presenilins in neurodegeneration independent of amyloid cascade [124,125]. However, given the fundamental function of Notch, it is hard to exclude that the phenotypes in conditional PS knock-out mice is due to the impaired Notch signaling. In the moss *Physcomitrella patens* (*P. patens*), which lacks Notch signaling, presenilin-deficit phenotype could be rescued by wild type presenilins as well as PS mutants without γ-secretase activity, indicating other functions of PS beyond γ-secretase activity [126]. Moreover, presenilins-knock-out mice exhibited more severe somite phenotype than mice lacking canonical Notch-signaling and mice deficient of Nicastrin, Aph-1 or Pen-2, which could still develop anterior somite [127]. In summary, all these clues point to the existence of independent functions of presenilins beyond γ-secretase.

**Figure 4:**
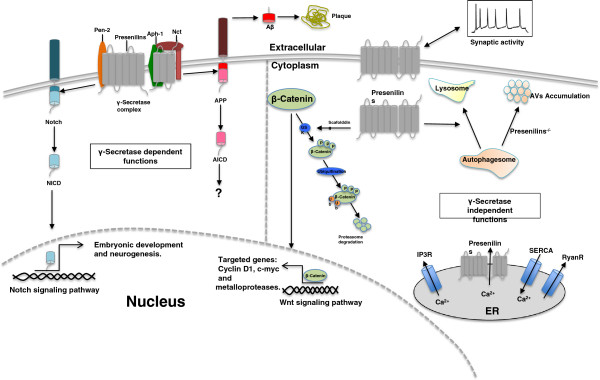
**Presenilin functions.** This diagram divides the functions of presenilins into γ-secretase-dependent and γ-secretase-independent ones by the vertical dash line. The γ-secretase activity of presenilins are exemplified by APP processing and Notch processing. Other γ-secretase independent functions of presenilins include stabilizing β-catenin in Wnt signaling pathway, regulating calcium homeostasis and its interaction with synaptic transmission. APP, amyloid β precursor protein; AICD, APP intracellular domain; NICD, Notch intracellular domain; Nct, Nicastrin; IP3, Inositol trisphosphate receptor; SERCA, Sarco/endoplasmic reticulum Ca^2+−^ATPase; Ryan R, Ryanodine receptor; GSK, Glycogen synthase kinase.

FAD-associated presenilins mutations exhibit not only significant heterogeneity on clinical features like age of onset, neurological and psychiatric symptoms, but also on neuropathology including greater NTF formation, altered neuritic plaque composition, presence of Pick body, and neuropathological lesion in basal ganglia and brainstem [128-130]. It is also reported that presenilins are involved in Wnt signaling, cell adhesion, calcium homeostasis, protein degradation and apoptosis, raising the possibility that γ-secretase-independent function of presenilins might contribute to the presenilins mutations-associated heterogeneity. The subsequent sections of this review will focus on these issues (Figure 4).

## PS1 and β-catenin

### β-Catenin in Wnt-signaling and cell-cell adhesion

β-Catenin is a signal transducer protein in Wnt-signaling pathway as well as a cell adhesion molecule [131]. β-Catenin carries out two distinct functions according to its cellular location: the membrane β-catenin forms complex with E-cadherin as cell-cell adhesion molecule; whereas the cytoplasmic β-catenin is involved in Wnt-signaling pathway to regulate gene expression. In the absence of Wnt ligand, β-Catenin undergoes phosphorylation by Glycogen Synthase Kinase-3β (GSK3β) with the assistance of Axin/APC complex, and then the phosphorylated β-catenin is constitutively degraded in ubiquitin proteasome pathway. Binding of Wnt to its receptor Frizzled and co-receptor LRP5/6 blocks phosphorylation of β-catenin by GSK3β, precluding the degradation of β-catenin. β-catenin is translocated into nuclear to activate transcriptional factor like T-cell-specific transcriptional factor 1 (TCF), to regulate target genes like cyclin D1, c-myc, and metalloproteases. On the other hand, membrane β-catenin acts as a bridge to link cadherins to α-catenin, and the latter binds to actin network, to stabilize adherence junctions as well as the cytoskeleton [131].

### PS1/β-catenin interaction and AD

PS1 negatively regulates β-catenin level via physically interacting with β-catenin through the cytosolic-loop structure of PS1 [132-136]. This function of PS1 is γ-secretase-independent since the D257A mutant could rescue the turnover of β-catenin as wild type PS1 does [137]. Though evidence supports the idea that PS1 works as a scaffold to facilitate β-catenin phosphorylation, the underlying mechanism remains to be elucidated [136-138].

It has been reported that the stabilization of β-catenin contributes to the development of skin cancer in PS1-deficient mice [139]. However, the precise role of β-catenin in AD pathogenesis is not clear. Most studies on sporadic AD indicated that reduced Wnt/β-catenin signaling might make a contribution to AD pathogenesis [140]. However, the studies on FAD were conflicting, some PS mutations were found to stabilize β-catenin while others destabilized it [132,136,137,141-143].

## Presenilins and calcium regulation

### Interaction of presenilins and calcium channels

Presenilins mutations have been reported to connect with abnormal intracellular calcium signaling, and presenilins mutations promote the release of Ca^2+^ from overloaded ER stores through IP3 receptor [144]. Presenilins could interact with IP3 receptors to regulate IP3 channel activity. Using patch-clamp techniques, PS1_M146L_ and PS2_N141I_ prolonged IP3 channel opening and increased Ca^2+^ leak permeability [145]. Studies on primary neurons from PS1_M146V_-expressing mice revealed that mutant PS1 increased the expression and recruitment of ryanodine receptor (RyanR) to regulate the IP3R Ca^2+^ signaling in primary neurons [146-148]. Apart from IP3 receptor and RyanR, prensenilins were also reported to interact with sarco-/endoplasmic reticulum Ca^2+^ ATPase (SERCA) to regulate intracellular calcium signaling [149]. These studies suggested that presenilins and their mutants regulate intracellular Ca^2+^ signaling via interacting with various calcium channel-related proteins.

### Presenilins themselves as Ca2+ leak channels

Presenilins themselves were reported to function as low-conductance, passive ER Ca^2+^ leak channels, which is independent of γ-secretase activity [150]. Many FAD mutants (e.g. PS1_M146V_ and PS2_N141I_) disrupt or abolish the Ca^2+^ leak channel activity, leading to overload of Ca^2+^ in ER [151]. It has been reported that presenilin transmembrane domain 7 and 9 contribute to the forming of the ion conductance pore, and transmembrane water-filled catalytic cavity of presenilin constitutes the Ca^2+^ leak channel [152]. However, Ca^2+^ channel function of presenilins has been challenged by another group, showing that FAD PS mutants regulate calcium level by regulating IP3R channel gating [145,153]. Further studies on the crystal structure of presenilin would be helpful to elucidate whether presenilins themselves act as Ca^2+^ channels.

### Dysregulation of autophagy in AD and PS-associated calcium abnormality

The accumulation of autophagic vacuoles (AVs) has been observed in dystrophic neurites around the amyloid plaques for decades [154-156]. Autophagy serves as cellular processing for dysfunctional cellular organelles and toxic protein degradation, important for cell survival under stress like nutrient deprivation. Autophagy mainly involves two steps: generation of autophagosome containing dysfunctional cellular organelles and degradation of the contents via fusing with lysosome or late endosome [157,158].

It has been well established that PS deficiency impairs the turnover of long-lived proteins like telencephalin(TLN) and α-synuclein [159,160], which results from the lysosome fusion failure and autophagy deficit. In PS1^−/−^ hippocampal neurons, TLN is accumulated in intracellular membrane organelles containing Apg12p and LC3, the autophagic vacuole markers, in both ultrastructure and immunostaining experiments [159,161]. The accumulation of TLN could be rescued by PS1_WT_, FAD-associated PS1 mutants or dominant-negative PS1 mutant (PS1_D257A_), indicating that the PS-associated autophagic vacuoles accumulation was independent of γ-secretase activity. The accumulation of autophagic vacuoles may be caused either by increased production of autophagic vacuoles, resulting from accelerated autophagy activity; or by reduced consumption, resulting from dysfunctional fusion with the lysosome/endosome. Given that the formation of TLN-positive autophagosome triggered by microbeads was normal, the authors proposed that observed accumulation of TLN-positive autophagic vacuoles was correlated with failed lysosome fusion, which was in accordance with mounting evidence supporting lysosome deficit as the underlying cause of the autophagy deficit in AD [160,162].

Other hypotheses explaining PS1-related autophagic vacuole accumulation involves the lysosome acidification deficiency. Lee et al. reported that PS1 acted as chaperone protein to facilitate the glycosylation of V-ATPase subunit V0a1, which helped V-ATPase traffic to lysosome and completed lysosome acidification [163]. The failed acidification of lysosome in PS1^−/−^ blastocyst-derived cell line (BD15) repressed the fusion of lysosome with intermediate AVs, resulting in accumulation of AVs. However, later studies argued that lysosome acidification appeared to be unimpaired in PS1^−/−^/PS2^−/−^ stem cells and the glycosylation of V0a1 subunit was unaffected. Nevertheless, Coen et al. demonstrated that the calcium loading of lysosome in PS1^−/−^ or PS1^−/−^/PS2^−/−^ cells was significantly less than wild type cells, which could be rescued by PS1 mutant without γ-secretase activity, indicating the γ-secretase-independent property. Given that PS1 itself could act as ER Ca^2+^ leak channels, they proposed that the accumulation of autophagic vacuoles often observed in AD could be interpreted by impaired PS1-related calcium abnormality [164-166].

It is well known that PS deficiency is related with AV abnormality [160,165], however, the relationship between autophagy and FAD-associated PS1 mutations is not well defined. For example, Esselens et al. reported that FAD-associated PS1 mutations rescued PS1 deficiency-related TLN-positive autophagy deficit; whereas Lee et al. reported autophagy deficit in PS FAD mutations human fibroblast. Thus, it needs further investigation to clarify the contribution of FAD-associated PS1 mutations to autophagy deficit of AD.

### Correlation of Cotton wool plaques (CWP) and abnormal Calcium signaling

CWP are large, non-cored and diffuse amyloid plaques, which are composed primarily of Aβ42 without surrounding neuritic dystrophy and glial activation in Alzheimer cases [167]. CWP is often associated with spastic paraparesis (SP) [168], both of which were reported in a subset of PS1 mutants like PS1_M233T_, PS1_R278T_ and PS1_ΔE9_[169,170]. The mechanism underlying these unique clinical and pathological phenotypes is unknown. It is well established that Ca^2+^ release from intracellular stores is increased in both sporadic and familial AD [171-173], and thus it is proposed that the disturbed Ca^2+^ regulation in FAD is correlated with CWP [174,175]. Over 20 PS1 mutations have been analyzed and though all PS1 mutations show increased Aβ42/40 ratio, their effects on calcium signaling are various. It’s very illuminating to correlate calcium dysfunction with FAD variant phenotypes, but the underlying mechanism needs further investigation.

### Presenilins and synaptic transmission

Another pathological aspect of Alzheimer’s Disease is the failure of synaptic transmission and further disturbance in the neural circuit. Many believe that independent of plaque formation, impairment of synaptic function is what accounts for AD pathogenesis [176]. It has been reported that Aβ plays an important role in maintaining efficient synaptic transmission and stabilizing the neural circuit [30,177]. Recently presenilins stand out to be a candidate participating in the release of neurotransmitter and synaptic scaling independent of their γ-secretase function. It was reported that presenilins are essential for regulating neurotransmitter release like glutamate [178]. Presynaptic knockout of presenilins leads to inhibition of theta burst-induced long-term potentiation. Moreover, the inhibition effect is probably mediated by depletion of endoplasmic reticulum Ca^2+^ storage and blockade of intracellular Ca^2+^ release [178]. PS1 was also proposed to regulate homeostatic synaptic scaling [179]. PS1 knockout and PS_M146V_ neurons fail to scale up synaptic strengths in response to tetrodotoxin treatment, which can be rescued by viral expression of wild type PS1. Furthermore, γ-secretase inhibitor does not influence the effect of presenilins on synaptic scaling, suggesting that this function is independent of γ-secretase in AD pathogenesis. On the other hand, synaptic activity can in turn modulate the activity of PS1 such as regulating Aβ40/42 ratio via altering PS1 conformation, thereby forming bidirectional interaction [180]. Using a Cer-PS1-Cit FRET sensor, the group discovered that spike bursts trigger PS1 conformational change through vesicle exocytosis. More importantly, the conformational change of wild type PS1 upregulates Aβ40/42 ratio, which is uniformly decreased in almost all cases of FAD mutations. Overall, mounting evidence points to a role of presenilins in synaptic transmission. However, the underlying mechanism is still not clear. The comprehensive interaction between presenilins and synaptic activity could result from presenilin’s functions independent and/or dependent of γ-secretase activity.

## Conclusion

γ-secretase sequentially processes its substrates at ϵ- and γ-sites and the enzymatic activities on two cleavages are distinct. As the catalytic component of the γ-secretase complex, FAD-associated presenilins affect γ-secretase activity on the γ-site but the effects on ϵ-cleavage vary. These studies suggest the possibility of development of γ-secretase modulators sparing the Notch signaling in the future. It has long been observed that presenilins are involved in functions independent of the γ-secretase activity, like interaction with β-catenin/Wnt signaling, calcium regulation and autophagy degradation. However, its contribution to AD pathogenesis is not clear. Further studies are needed to clearly define the function of presenilins and its role in AD pathogenesis.

## Abbreviations

AD: Alzheimer’s disease; PS: Pensenilin; APP: amyloid β precursor proteins; BACE1: Beta-site APP cleaving enzyme 1; Aβ: Amyloid β protein.

## Competing interest

The authors declared that they have no competing interest.

## Authors’ contributions

SZ carried out literature search and drafted the manuscript. MZ wrote one section and critically revised the manuscript. FC drafted one of the figures and provided comments for the manuscript. WS was the supervisor of the research group, provided the guidance and instructions and critically revised the manuscript. All authors read and approved the final manuscript.
